# Supplementary material for: Graphene Film Growth on Silicon Carbide by Hot Filament Chemical Vapor Deposition
Source: Nanomaterials (Basel). 2022 Sep 1;12(17):3033. doi: 10.3390/nano12173033 (PMC9458213; doi:10.3390/nano12173033)
Supplement: Supplementary file 1 [file nanomaterials-12-03033-s001.zip › nanomaterials-1860114-supplementary.pdf]

Graphene Film Growth on Silicon Carbide by Hot Filament Chemical Vapor Deposition

Sandra Rodríguez-Villanueva, Frank Mendoza, Brad R. Weiner and Gerardo Morell

Supplementary information

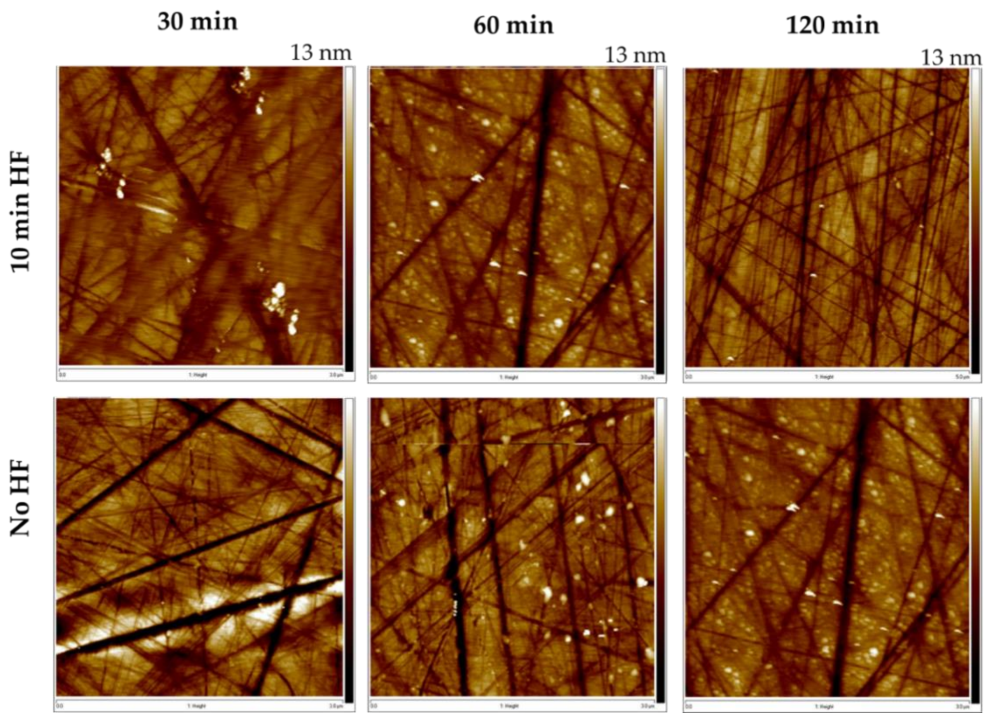

Figure S1. AFM measurements of SiC substrates cleaned and uncleaned at different annealing time (30, 60 and 120 minutes).
